# A homeostatic mechanism rapidly corrects aberrant nucleocytoplasmic ratios maintaining nuclear size in fission yeast

**DOI:** 10.1242/jcs.235911

**Published:** 2019-11-14

**Authors:** Helena Cantwell, Paul Nurse

**Affiliations:** 1 Cell Cycle Laboratory, The Francis Crick Institute, London NW1 1AT, UK; 2 Laboratory of Yeast Genetics and Cell Biology, Rockefeller University, New York, NY 10065, USA

**Keywords:** Nuclear size control, Organelle scaling, Nucleus, Fission yeast, Homeostasis

## Abstract

Nuclear size scales with cell size across a wide range of cell types. The mechanism by which this scaling is maintained in growing cells remains unclear. Here, we investigate the mechanism of nuclear size homeostasis in the simple eukaryote fission yeast, by monitoring the recovery of aberrant nuclear volume to cell volume (N/C) ratios following perturbation. We demonstrate that both high and low N/C ratios correct rapidly, maintaining nuclear size homeostasis. We assess the kinetics of nuclear and cellular growth and of N/C ratio correction, and demonstrate that nuclear and cellular growth rates are not directly coupled. We propose that the mechanism underlying nuclear size homeostasis involves multiple limiting factors implicated in processes including nucleocytoplasmic transport, lipid biogenesis and RNA processing. We speculate that these link cellular size increases to changes in nuclear contents, which in turn lead to changes in nuclear membrane surface area. Our study reveals that there is rapid nuclear size homeostasis in cells, informing understanding of nuclear size control and size homeostasis of other membrane-bound organelles.

## Abstract (precis)

**Summary:** Nuclear size homeostasis is achieved by a rapid correction mechanism modulating nuclear growth rate in growing fission yeast cells.

## INTRODUCTION

Membrane-bound organelles increase in size as cells grow. However, the mechanisms ensuring organellar size and membrane growth are coordinated with cellular growth are not well understood. The nucleus is a useful model to investigate this important but understudied problem, as nuclei are generally simply shaped, easily measured, and present in single copy. Nuclear size scaling with cellular size has been observed in a wide range of cell types (Boveri, 1905; Gregory, 2005; Jorgensen et al., 2007; Levy and Heald, 2010; Maeshima et al., 2011; Neumann and Nurse, 2007; Robinson et al., 2018). In the budding and fission yeasts, it has been proposed that nuclear volume (*V*_nuc_) scales with cellular volume (*V*_cell_) (Jorgensen et al., 2007; Neumann and Nurse, 2007) and in the case of fission yeast, a nuclear to cellular volume (N/C) ratio of 8% is maintained over a 35-fold range of cell volumes. Although recent studies have identified some molecular players and biological processes with roles in nuclear size control (Brownlee and Heald, 2019; Cantwell and Nurse, 2019; Jevtić et al., 2019; Kume et al., 2019, 2017), it is not known how these are integrated in a global mechanism to maintain nuclear scaling in individual cells as they grow and divide. Investigation of this mechanism requires understanding of the kinetics of this process, in particular of how individual cells with aberrant N/C ratios correct towards the population mean and what this implies for the nuclear size control mechanisms employed by cells.

By perturbing the N/C ratio of fission yeast cells, and monitoring resultant nuclear and cellular growth rates, we show here that N/C ratio homeostasis is operative in growing fission yeast cells, and we probe this homeostatic mechanism by assessing the kinetics of N/C ratio recovery and how both high and low N/C ratios correct.

## RESULTS AND DISCUSSION

### Nuclear to cellular volume ratio is maintained through interphase, in multinucleate cells and between fission yeast species

**Fig. 1.**
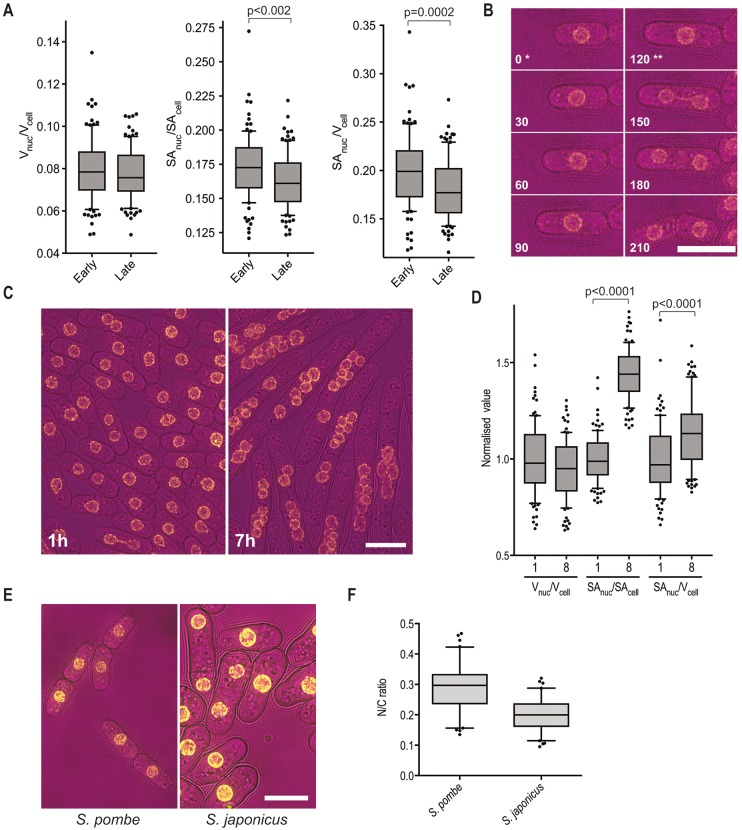
***V*_nuc_/*V*_cell_ is maintained through interphase, in multinucleate cells and between related fission yeast species.** (A) The nucleus to cell volume (*V*_nuc_/*V*_cell_), nuclear to cellular surface area (*SA*_nuc_/*SA*_cell_) and nuclear surface area to cell volume (*SA*_nuc_/*V*_cell_) ratio of wild-type cells early and late in interphase. *n*=100 cells. Wild-type cells were measured through time-lapse microscopy (the nuclear envelope marker was Cut11–GFP) at 25°C and 5 min intervals. Each cell was measured early and late in interphase. Early, time point of first image following cell division of parent cell. Late: time point before first image displaying a Cut11–GFP focus. Cut11 is the *S. pombe* Ndc1 orthologue that transiently associates with the spindle pole body (SPB) during mitosis (West et al., 1998). (B) Representative cell from time lapse described in A. Time (min) following the early interphase time point indicated. Bright field, magenta; Cut11–GFP, yellow. *Early interphase. **Late interphase. (C) Representative images of *cdc11-119* cells grown at 25°C then incubated at 37°C for time indicated. Bright field, magenta; Cut11–GFP, yellow. (D) *V*_nuc_/*V*_cell_, *SA*_nuc_/*SA*_cell_ and *SA*_nuc_/*V*_cell_ ratios of mononucleate (labelled 1) and octonucleate (labelled 8) *cdc11-119* cells. Ratios were normalized to the mean ratio of mononucleate population. *n*=100 cells/condition. For both mononucleates and octonucleates, the *V*_nuc_/*V*_cell_, *SA*_nuc_/*SA*_cell_ and *SA*_nuc_/*V*_cell_ ratios displayed were calculated from measurements of the same 100 cells. (E) Representative images of *S. pombe* and *S. japonicus* cells (32°C). Brightfield, magenta; Cut11–GFP, yellow. (F) Interphase N/C ratio of *S. pombe* and *S. japonicus* cells grown at 32°C (*n*=80 cells/species). Box plots represent the 25–75th percentiles, and the median is indicated. The whiskers show the 10–90th percentiles. Paired (A) or unpaired (D,F) Student's *t*-tests were used to calculate *P*-values. Scale bars: 10 µm.

Different aspects of membrane bound organelles could scale with cell size, such as linear dimensions, surface area or volume. Nuclear volume and nuclear membrane surface area of *Schizosaccharomyces pombe* fission yeast cells were monitored by time lapse microscopy, measuring nuclear and cellular volumes (*V*) and surface areas (*SA*) at the beginning and end of interphase (Fig. 1A,B). The *V*_nuc_/*V*_cell_ ratio was the same at the two time points, confirming results from an earlier pilot study of five cells (Neumann and Nurse, 2007), but both *SA*_nuc_/*SA*_cell_ and *SA*_nuc_/*V*_cell_ ratios were significantly altered, suggesting that nuclear size homeostasis operates through a *V*_nuc_/*V*_cell_ mechanism. This was tested further using multinucleate cells where nuclear surface area and volume diverge more dramatically than in mononucleates. At the restrictive temperature, *cdc11-119* cells undergo repeated nuclear division without septation, giving rise to multinucleate elongated cells (Nurse et al., 1976) (Fig. 1C). Both *SA*_nuc_/*SA*_cell_ and *SA*_nuc_/*V*_cell_ ratios were significantly higher in octonucleates than in mononucleates, whereas the *V*_nuc_/*V*_cell_ ratio was not significantly different between mononucleates and octonucleates (Fig. 1D). In addition, the *V*_nuc_/*V*_cell_ ratio appears to be conserved between *S. pombe* and *Schizosaccharomyces japonicus*, which are predicted to have diverged evolutionarily ∼200 million years ago (Rhind et al., 2011). Their interphase *V*_nuc_/*V*_cell_ ratios were similar (Fig. 1E,F), despite divergent cell sizes and mitotic strategies, and significant evolutionary divergence (Gu et al., 2012).

### Aberrant *V*_nuc_/*V*_cell_ ratios undergo rapid correction

**Fig. 2.**
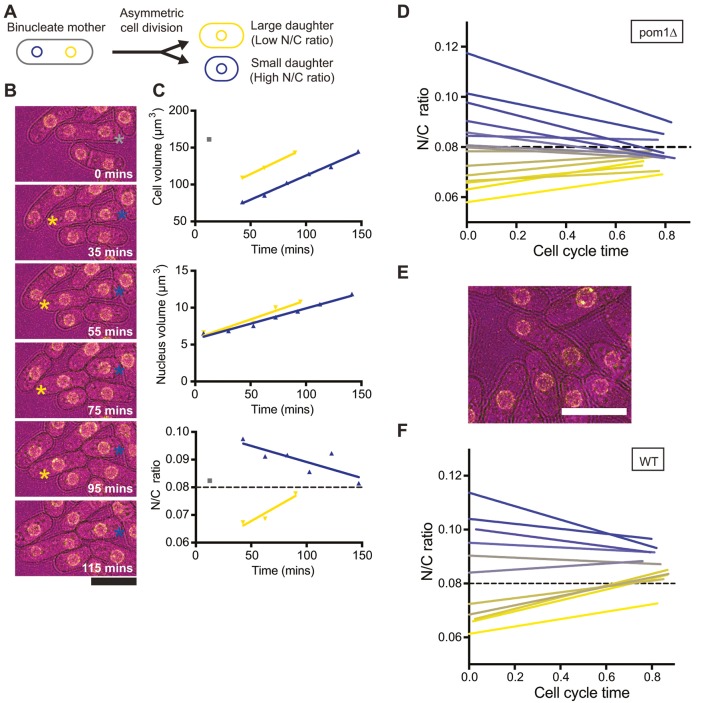
**Rapid N/C ratio homeostasis observed in cells ‘born’ with aberrant N/C ratios.** (A) Schematic of asymmetric cell division of a *pom1*Δ cell producing large (low N/C ratio) and small (high N/C ratio) daughters. (B) Images from time-lapse microscopy (32°C) of a representative pair of cells. Bright field, magenta; Cut11–GFP, yellow. Binucleate mother (grey), large (yellow) and small (blue) daughters are indicated. (C) Cell volume, nucleus volume and N/C ratio of cells in B. Cells were measured every 5 min from mother cell nuclear division to daughter cell nuclear division (assigned into 20 min bins). Colours as in B. (D) N/C ratio against cycle time for *pom1*Δ cells (32°C, *n*=156 cells). Cells were assigned into cohorts according to the initial N/C ratio (first time bin) (*n*≥10 cells per cohort). Linear regression lines are shown. (E) Representative wild-type cell that had undergone asymmetric nuclear division. Bright field, magenta; Cut11–GFP, yellow. (F) N/C ratio against cycle time for wild-type cells ‘born’ with aberrant N/C ratios (25°C, *n*=44). Cells were assigned into cohorts according to the initial N/C ratio (four cells/cohort). Regression lines are shown. Dashed lines represent the wild-type N/C ratio. Scale bars: 10 µm.

Having established that a constant nuclear to cellular volume (N/C) ratio is maintained, we sought to investigate the mechanism responsible for this homeostasis by assessing the response of individual cells to N/C ratio perturbation. *Pom1*Δ cells undergo symmetric nuclear division but asymmetric cell division. More than 80% of *pom1*Δ cells, compared with less than 5% of wild-type, divide asymmetrically (Bahler and Pringle, 1998). Consequently, *pom1*Δ cells exhibit a large range of birth N/C ratios. Time lapse microscopy was carried out, and the nuclear and cellular volume of individual cells displaying asymmetric cell division was measured every five minutes from nuclear division of the mother cell to nuclear division of the daughter cell. From this data, the N/C ratio was calculated for 156 cells (Fig. 2A–C). Birth N/C ratios had a mean value of 0.084, like wild-type cells, but that ratio was very variable, ranging from 0.053 to 0.132. The asymmetric cell divisions of *pom1*Δ cells lead to a range of cell cycle durations, so data was cycle time normalised. The 156 cells were assigned into cohorts according to the initial N/C ratio (N/C ratio of first 20 min time bin). The different N/C ratio cohorts of cells displayed rapid homeostasis towards the wild-type N/C ratio value (0.08), generally within one cell cycle (Fig. 2D). This experiment was repeated in wild-type cells where altered N/C ratios occur rarely, usually as a result of asymmetric nuclear division (Fig. 2E), and so fewer aberrant ratio cells could be analysed. However, like *pom1*Δ cells, they exhibited N/C ratio homeostasis (Fig. 2F).

### Nuclear and cellular growth rates are not directly coupled

**Fig. 3.**
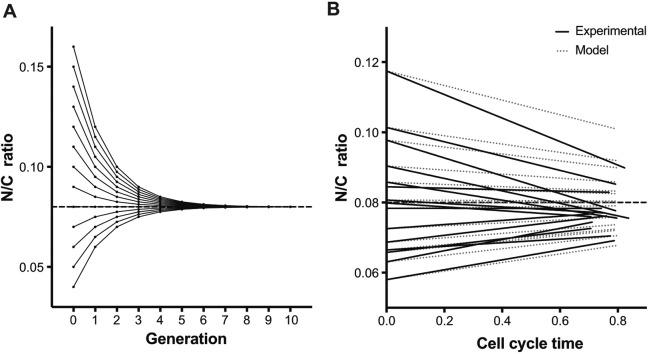
**Nuclear and cellular growth rate are not directly coupled across the population.** (A) Simulated N/C ratios of cells with initial N/C ratios ranging from 0.04 to 0.16 over 11 generations. Cell volume doubles between successive generations. Nuclear growth rate (*GR*_nuc_) is maintained at 8% of cellular growth rate (*GR*_cell_). (B) Comparison of experimental data for cohorts of *pom1*Δ cells described in Fig. 2D (black), spanning ∼0.8 cell cycles for each cohort, to N/C ratio change predicted over 0.8 cell cycles by the model (grey dotted) in which *GR*_nuc_ is maintained at 8% of *GR*_cell_. The dashed lines represent the wild-type N/C ratio.

The kinetics with which N/C ratios recover from perturbation are informative about the mechanisms by which nuclear size homeostasis is achieved as different mechanisms will lead to different recovery times. Therefore, we analysed the kinetics of the N/C ratio recovery to allow us to distinguish between potential models. The simplest model by which N/C ratio homeostasis could be achieved is direct coordination of nuclear and cellular growth rates. If nuclear growth rate was maintained at 8% of cellular growth rate then N/C ratio values would converge on a value of 0.08. Changes to N/C ratios with an initial range of 0.04 to 0.16 were simulated and demonstrate homeostatic behaviour; N/C ratios eventually converged on a wild-type value of 0.08 but only after around four generations (Fig. 3A). The experimental data in Fig. 2 was compared to modelled values. Experimental and modelled linear regression lines of N/C ratio of cohorts of cells over ∼0.8 of a cell cycle were compared (Fig. 3B). For seven of eight high N/C ratio cohorts, and five of seven low N/C ratio cohorts, the N/C ratio recovery towards the population mean observed experimentally was more rapid than that predicted by the model.

### A rapid control mechanism corrects aberrant N/C ratios

**Fig. 4.**
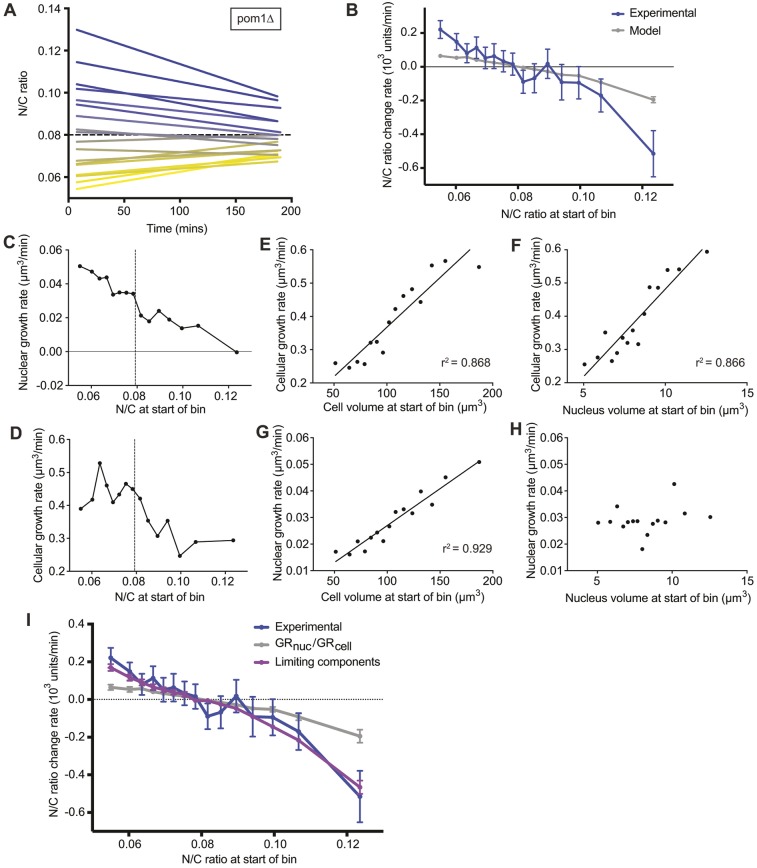
**Cells with more-aberrant N/C ratios correct more rapidly.** (A) N/C ratio of *pom1*Δ cells (25°C) measured during the 200 min after septation (5 min intervals, 20 min bins) (*n*=180 cells). The dashed line represents wild-type N/C ratio. Data were assigned into cohorts according to the initial bin N/C ratio (10 cells/cohort). Linear regression of cohort N/C ratio coloured by cohort average initial N/C ratio (blue, high; grey, intermediate; yellow, low). (B) Experimental (blue) and modelled (grey) mean N/C ratio change rate (with 95% confidence intervals) of individual 20 min time bins from *pom1*Δ data described in A assigned into cohorts according to the N/C ratio at the start of the bin. Modelled data calculated from experimental cell and nuclear volumes at the start of bins and cellular growth rates assuming nuclear growth rate is 8% of cellular growth rate. (C,D) Mean nuclear growth rate (C) and cellular growth rate at the start of the bin (D) of individual 20 min time bins from data described in A assigned into cohorts according to the N/C ratio at start of bin. The dashed line represents the N/C ratio at the start of the bin at which no N/C ratio change is observed. (E–H) Cellular growth rate (E,F) and nuclear growth rate (G,H) of individual time bins from data described in A assigned into cohorts according to the cell (E,F) or nucleus (G,H) volume at the start of the bin. Linear regression lines are shown except for H as *r*^2^<0.2. (I) Experimental (blue), limiting components model (see Materials and Methods) (purple) and *GR*_nuc_/*GR*_cell_=0.08 model (grey) mean N/C ratio change rate (with 95% confidence intervals) of individual 20 min time bins. Time bins of *pom1*Δ data described in Fig. 4 are assigned into cohorts according to the N/C ratio at the start of the bin.

To test the homeostatic mechanism further, the kinetics of N/C ratio change during interphase were examined in more detail. Nuclear and cellular volumes of *pom1*Δ cells were assessed for a larger number of cells using a lower temperature (25°C), allowing greater time resolution. Cells and nuclei were measured for 200 min from the septation of the mother cell, or until nuclear division if this was sooner, and instantaneous growth rates calculated. A range of initial N/C ratios from 0.049 to 0.164 was observed (Fig. 4A). The kinetics of N/C ratio homeostasis and the parameters affecting the kinetics in individual cells at specific times as they correct, were analysed. For each 20 min time bin, cellular growth rate, nuclear growth rate and the N/C ratio change rate were calculated and assigned to a cohort according to the N/C ratio at the start of the bin. This indicated that correction towards an N/C ratio of 0.079 occurred, and that the more aberrant an N/C ratio was, the more rapidly it corrected (Fig. 4B). Experimental N/C ratio change rate values were compared to modelled values assuming direct coordination of nuclear growth rate and cellular growth rate (Fig. 4B). The experimental mean was not within 95% confidence intervals of the modelled mean in any of the 15 cohorts. Cells with both high and low N/C ratios, exhibited an N/C ratio change rate faster in the experimental than in the modelled data. Therefore, a homeostatic mechanism is operative, which is more rapid than simple coordination of nuclear and cellular growth rates, although it is possible that coordination of nuclear and cellular growth rates might form part of the N/C ratio homeostasis mechanism.

### Nuclear growth rate correlates with cell volume, not nuclear volume

The relationships between growth rates and parameters of the cells were further assessed to gain understanding of how N/C ratio homeostasis is achieved. Average cellular and nuclear growth rates were calculated for cohorts of time bins assigned according to the N/C ratio at the start of the bin (Fig. 4C,D). Nuclear growth rate correlated near-linearly with the N/C ratio at the start of the bin in cells with both low and high N/C ratios, decreasing with increasing N/C ratio (Fig. 4C). Nuclear growth rate intercepted the *x*-axis at an N/C ratio ∼50% higher than the N/C ratio at which no correction is observed (0.079) suggesting that, at very high N/C ratios, nuclear growth ceases. Average cellular and nuclear growth rates were calculated for cohorts of time bins assigned into cohorts by nucleus or cell volume at the start of the bin. Cellular growth rate correlated with both the cell (Fig. 4E) and nucleus (Fig. 4F) volume at the start of the bin. Nuclear growth rate correlated with the cell volume at the start of the bin (Fig. 4G) but did not correlate with nucleus volume at the start of the bin (Fig. 4H). Therefore, nuclear growth rate was not determined by nuclear volume but instead correlated with cell volume and negatively with N/C ratio.

### The kinetics of recovery observed are similar to those predicted by a limiting components model of nuclear size control

The observation that at very high N/C ratios (>0.12) nuclear growth rate is much decreased (Fig. 4C) suggested an alternative possible model for N/C ratio homeostasis. At N/C ratios above 0.12, nuclear growth may be prevented because a factor, or factors, required for nuclear growth becomes limiting, giving rise to a limiting components model (Goehring and Hyman, 2012; Marshall, 2015). In such a model, the amount of a component required for nuclear growth would be determined by cell volume. Nuclear growth rate would scale with the free cellular concentration of the component. In large cells with low N/C ratios, the component would not be limiting, so nuclear growth rate would be high, decreasing as the pool of excess component was depleted. In cells with mid-range N/C ratios, nuclear growth rate would generally scale with cellular growth rate, as the pool of limiting component was replenished by cellular growth. In small cells with extremely high N/C ratios, the free cellular concentration of the limiting component would rapidly become lower than that required for nuclear growth, so nuclear growth would reduce or even cease.

The kinetics of N/C ratio change predicted by such a limiting components model (Materials and Methods) were compared to experimental data and to those predicted by the alternative model described above in which nuclear and cellular growth rates are directly coupled (Fig. 3). The mean N/C ratio change rate was calculated for time bins assigned into cohorts according to the N/C ratio at the start of the bin (Fig. 4I). The limiting components model describes the experimental data well and predicts N/C ratio correction towards a value of ∼0.08. The modelled mean N/C ratio change rate is within 95% confidence intervals of the experimental mean in 14 of 15 N/C ratio cohorts. Therefore, the N/C ratio homeostasis observed in *S. pombe* cells is consistent with a limiting components model in which concentrations of nuclear components limit the nuclear growth rate. As a consequence, a nucleus in a low N/C ratio cell will undergo percentage size increase faster than a nucleus in a cell with a normal N/C ratio. The inverse is true for nuclei in high N/C ratio cells, as the percentage size increase would be slower than in cells with normal N/C ratios, giving rise to nuclear size homeostasis. A limiting component determining nuclear size has been proposed previously (Goehring and Hyman, 2012), and assumes that the amount of a factor required for nuclear growth is directly proportional to cell volume. This is reasonable given that biosynthesis rate (Schmoller and Skotheim, 2015) and protein amount (Crissman and Steinkamp, 1973; Newman et al., 2006; Williamson and Scopes, 1961) generally scale with cell size. In the case of nuclear size homeostasis, the amount of the limiting factor or factors in the cytoplasm determines nuclear volume growth. Increase in nuclear volume requires an increase in nuclear membrane surface area. As we have shown that nuclear membrane surface area does not scale simply with cell volume, we propose that the primary driver of nuclear size homeostasis is the amount of nuclear content, largely determined by the balance between nuclear import and export, and that changes in the nuclear membrane surface area are brought about indirectly by the nuclear contents. This is consistent with the observation that inhibition of nuclear export results in nuclear expansion (Neumann and Nurse, 2007) and, speculatively, might involve a pressure-sensing mechanism acting at the membrane. Our screens of fission yeast non-essential and essential gene deletion mutants for those with aberrant nuclear size phenotypes have implicated a range of factors and biological processes as being involved in this process, including nucleocytoplasmic transport, lipid biogenesis, LINC complexes and RNA processing in nuclear size control (Cantwell and Nurse, 2019; Kume et al., 2017). Thus, there could be multiple limiting components involved in the nuclear size homeostatic mechanism.

## MATERIALS AND METHODS

### Strains and growth conditions

*S. pombe* media and methods used were as described previously (Moreno et al., 1991) and cells were grown in YE4S medium. Fission yeast strains used in this study are listed in Table S1.

### Imaging and image analysis

Fluorescence imaging was carried out using a DeltaVision Elite microscope (Applied Precision) comprising an Olympus IX71 wide-field inverted fluorescence microscope, an Olympus Plan APO 60×, 1.4 NA oil objective and a Photometrics CoolSNAP HQ2 camera (Roper Scientific) in an IMSOL ‘imcubator’ Environment Control System. Images were acquired in 0.2 µm or 0.4 µm *z*-sections over 4.4 µm, with a bright-field reference image in the middle of the sample, and deconvolved using SoftWorx (Applied Precision). Representative images shown are maximum intensity projections of deconvolved images.

Imaging was carried out in liquid medium on glass slides at 25°C unless otherwise stated or, for time lapse microscopy, in a microfluidic chamber. Time lapse microscopy was carried out using a CellASIC ONIX Microfluidic Platform with Y04C microfluidics plates (Merck). Cells were imaged in the 3.5 µm and 4.5 µm height chambers in YE4S. 50 µl of exponentially growing cells (1.26×10^6^ cells/ml) was loaded into the plate at a flow rate of 8 psi, cells that were not trapped were washed out at a flow rate of 5 psi, then a flow rate of 3 psi was maintained for the duration of the experiment.

### Nuclear and cellular size measurement and *V*_nuc_/*V*_cell_ ratio, *SA*_nuc_/*SA*_cell_ and *SA*_nuc_/*V*_cell_ ratio calculation

Fluorescence images displaying nuclear envelopes were overlaid on bright-field images displaying cell outlines. The ImageJ Pointpicker plugin (NIH) was used to determine coordinates of points on the cellular and nuclear surfaces, then the distance between these points was calculated to determine cell length, cell width and nucleus axial and equatorial diameters. From these linear measurements, nuclear volume to cell volume (N/C) ratio, nuclear surface area to cellular surface area ratio (*SA*_nuc_/*SA*_cell_) and nuclear surface area to cellular volume (*SA*_nuc_/*V*_cell_) ratio were calculated assuming radial symmetries and approximating the cell as a rod and the nucleus as a prolate ellipsoid (Neumann and Nurse, 2007). For multinucleate cells, individual nuclei were measured, then their volumes or surface areas summed for calculation of whole-cell N/C, *SA*_nuc_/*SA*_cell_ and *SA*_nuc_/*V*_cell_ ratios. To measure the volume of each part of a septated cell (either side of the septum), the length from cell tip to septum (*L*) and cell width (*w*) were measured and volume approximated as a cylinder of diameter *w* and height [*L−*(*w*/2)] joined to a hemisphere with diameter *w.*

### Counting nuclei in multinucleate cells

Nuclei were counted in images of multinucleate cells using the Cell Counter plugin of ImageJ (NIH). All *z*-slices of each deconvolved image were analysed to ensure nuclei in all *z*-planes were counted.

### Statistical tests

Unless otherwise indicated, two-tailed unpaired Student's *t*-tests were used to determine significance of difference between two populations of N/C ratio measurements. Linear regression lines were fitted (and *r*^2^ goodness of fit values calculated) using Prism 7.0 default parameters.

### Limiting components model derivation

Limiting components models for organelle scaling have previously been proposed (Goehring and Hyman, 2012; Marshall, 2015). This model assumes that the limiting component is incorporated into the nucleus at a rate dependent on the concentration of the unincorporated component in the cell. Experimentally, we did not observe nuclear shrinkage; nuclei in cells with extremely high N/C ratio stopped growing but did not reduce in size. Based on this, a second assumption is made; it is assumed that the rate at which the limiting component is removed from the nucleus is 0, its incorporation is irreversible.

Where *T* represents the total number of limiting component units in the cell, *N* represents the number incorporated into the nucleus and *V*_cell_ represents cell volume, the concentration of free units of limiting component in the cell is given by 
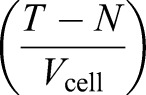
. Where *k*_on_ is the association rate constant for incorporation of units of component into the nucleus, the change in number of nucleus incorporated units of component per unit time 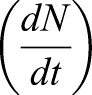 is given by:(1)
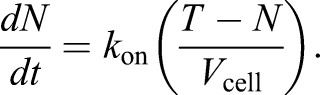
Assuming that *T* is directly proportional to cell volume, and *N* is directly proportional to nuclear volume, when α and β are constants, gives:(2)
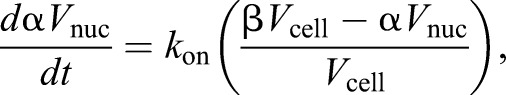
which can be rearranged to:(3)
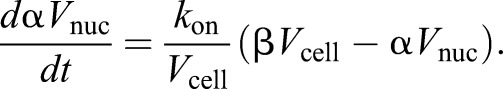
We know from experimental data (Fig. 4C) that when 
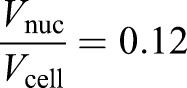
, 
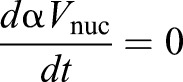
, so:
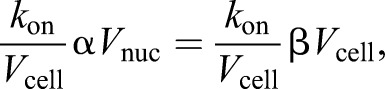


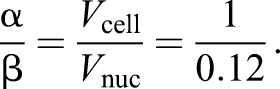


Combining this with Eqn 2 gives:
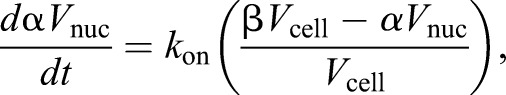

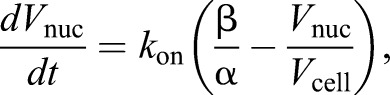

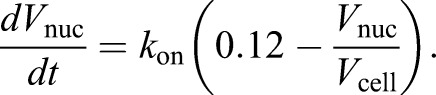


Therefore, this model predicts that there should be a linear correlation between nuclear growth rate 
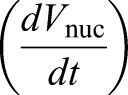
 and 
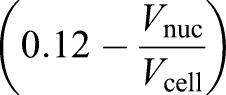
 with a gradient of *k*_on_. In the *pom1*Δ dataset described in Fig. 4, a linear correlation was indeed observed (*r*^2^=0.932), with the gradient of a regression line that passed through the origin being 0.73 (Fig. S1A). If *k*_on_ is 0.73:
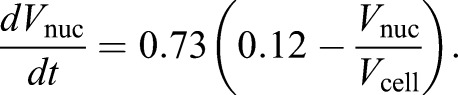
In order to determine whether this model gives rise to the kinetics of N/C ratio homeostasis observed experimentally, cellular growth rate must also be modelled. We observed experimentally that cellular growth rate correlated with both cell volume and nucleus volume (Fig. 4E and F). It is more likely that cellular growth rate is determined by cell volume than by nucleus volume. Here, cellular growth rate is assumed to be directly proportional to cell volume, so where γ is a constant:
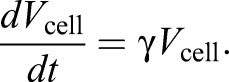


The value of γ can be determined from experimental data; it will be the gradient of a regression line of a graph of cellular growth rate 
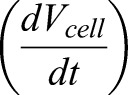
 plotted against cell volume (*V*_cell_) that passes through the origin (Fig. S1B). The experimental data from the *pom1*Δ dataset described in Fig. 4 gave γ=0.0036 so:
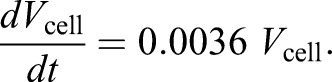


Therefore, this limiting components model predicts a nuclear growth rate (*GR*_nuc_) and cellular growth rate (*GR*_cell_) of:
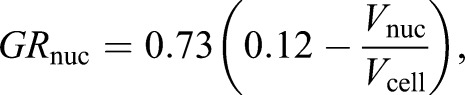
and:
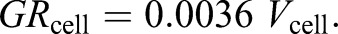


## Supplementary Material

Supplementary information
