# Supplementary material for: A homeostatic mechanism rapidly corrects aberrant nucleocytoplasmic ratios maintaining nuclear size in fission yeast
Source: J Cell Sci. 2019 Nov 14;132(22):jcs235911. doi: 10.1242/jcs.235911 (PMC6899009; doi:10.1242/jcs.235911)
Supplement: Supplementary information [file joces-132-235911-s1.pdf]

**Table S1:** Fission yeast strains used in this study.

| Strain | Species | Genotype | Origin |
|---|---|---|---|
| PN3779 | *S. pombe* | *cut11-GFP::ura4+ leu1-32 ura4-D18 h+* | Lab collection |
| PN3803 | *S. pombe* | *cdc11-119 cut11-GFP::ura4+ leu1-32 ura4-D18 h-* | Lab collection |
| NIG8181 | *S. japonicus* | *mat6j-2017 cut11-GFP::natR h+* | (Aoki et al., 2011) |
| PN10423 | *S. pombe* | *pom1Δ::ura4+ cut11-GFP::ura4+ h-* | Lab collection |

Aoki, K., Hayashi, H., Furuya, K., Sato, M., Takagi, T., Osumi, M., Kimura, A. and Niki, H. (2011). Breakage of the nuclear envelope by an extending mitotic nucleus occurs during anaphase in Schizosaccharomyces japonicus. *Genes Cells* 16, 911-926. doi:10.1111/j.1365-2443.2011.01540.x

Journal of Cell Science • Supplementary information

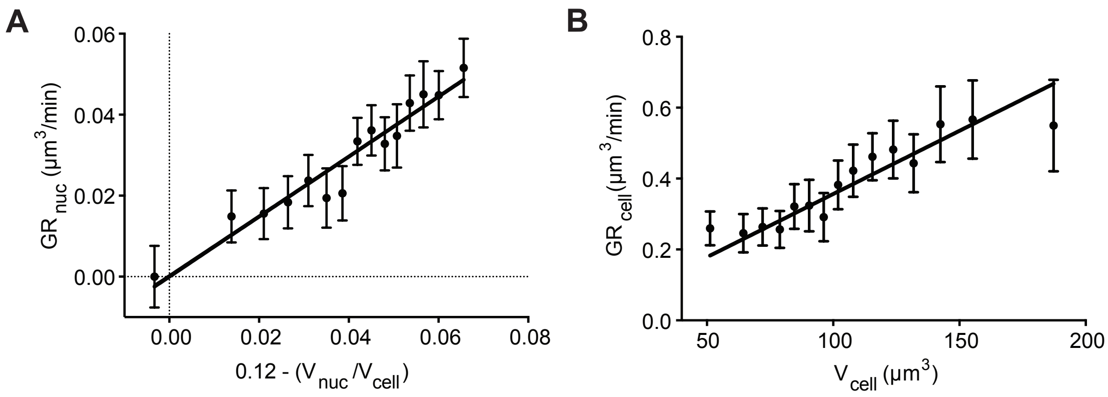


**Figure S1:** Data derived from *pom1*Δ dataset in Fig. 4. (A) Mean nuclear growth rate ($GR_{nuc}$) cohorted by $\left(0.12 - \frac{V_{nuc}}{V_{cell}}\right)$. (B) Mean cellular growth rate ($GR_{cell}$) cohorted by cell volume ($V_{cell}$). 95% confidence limits (15 cohorts, ≥100 time bins per cohort) and linear regression lines shown.
